# Stereotypies in autism: a video demonstration of their clinical variability

**DOI:** 10.3389/fnint.2012.00121

**Published:** 2013-01-03

**Authors:** Sylvie Goldman, Paul E. Greene

**Affiliations:** ^1^Saul R. Korey Department of Neurology, Albert Einstein College of MedicineBronx, NY, USA; ^2^Department of Pediatrics, Albert Einstein College of MedicineBronx, NY, USA; ^3^Rose F. Kennedy Intellectual and Developmental Disabilities Research Center, Albert Einstein College of MedicineBronx, NY, USA; ^4^Department of Neurology, Columbia University Medical CenterNew York, NY, USA

**Keywords:** autism, motor stereotypies, video, classification, variability

## Abstract

In autism, stereotypies are frequent and disabling, and whether they correspond to a hyperkinetic movement disorder, a homeostatic response aiming at sensory modulation, or a regulator of arousal remains to be established. So far, it has been challenging to distinguish among these different possibilities, not only because of lack of objective and quantitative means to assess stereotypies, but in our opinion also because of the underappreciated diversity of their clinical presentations. Herein, we illustrate the broad spectrum of stereotypies and demonstrate the usefulness of video-assisted clinical observations of children with autism. The clips presented were extracted from play sessions of 129 children with autism disorder. We conclude that compared to widely used questionnaires and interviews, systematic video observations provide a unique means to classify and score precisely the clinical features of stereotypies. We believe this approach will prove useful to both clinicians and researchers as it offers the level of detail from retrievable images necessary to begin to assess effects of age and treatments on stereotypies, and to embark on the type of investigations required to unravel the physiological basis of motor behaviors in autism.

## Introduction

Autism Disorder is a neurodevelopmental disorder defined clinically by the presence of qualitative impairments in three core domains: social interaction, communication, and restricted repetitive and stereotyped patterns of behavior, interests and activities. Repetitive, purposeless, patterned, rhythmic movement called motor stereotypies belong to the third criterion and as such are the only motor deficits included in the DSM-IV-TR (American Psychiatric Association, 2000) for a diagnosis of Autism. Following the new wave of biology-based research in autism, motor anomalies are receiving increasing attention. Indeed, until recently deficient sociability and language and communication, the other two DSM-IV-TR and ICD-10 WHO (World Health Organization, 1992) criteria, were the focus of most studies. This new focus is reflected in the proposed upcoming DSM-5 diagnostic criteria for Autism Spectrum Disorders (ASD) which would be reduced to two criteria: persistent deficits in social communication and social interaction across contexts, and restricted, repetitive patterns of behavior, interests, or activities. The second criterion includes not only rituals and routines but also repetitive movements and sensory impairments. The inclusion of motor and sensory symptoms will require clinicians to develop the proper skills to identify and characterize these core symptoms.

Stereotypies do not only occur in the context of a neurodevelopmental disorder (i.e., secondary stereotypies) like autism, blindness, or intellectual disability, they are also observed in typically developing infants (i.e., primary stereotypies); the latter usually subside around age 3 years (Thelen, 1979) whereas secondary stereotypies tend to persist through life in various forms. The persistent motor stereotypies of autism encompass a broad range of simple and complex typically bilateral movements. They involve one or multiple body parts and can have a twisting or circular quality. Their duration, frequency and intensity are variable. Especially in children with limited cognitive abilities it is rarely possible to assess reliably the suppressibility or the presence of a premonitory urge, which contributes to the difficulty differentiating them from tics. If frequent, stereotypies can interfere with learning and with the quality of social interactions. Stereotypies are stigmatizing and may evolve into self-injurious behaviors which represent a major concern for parents who spend time and money on occupational therapy to try to alleviate them.

Much controversy surrounds the causes of stereotypies, and while no model has obtained overwhelming support, the currently predominant behavioral theory Applied Behavioral Analytic Approach (ABA) suggests that stereotyped movements are maintained by reinforcement associated with either automatic reinforcement or social interactions (Cunningham and Schreibman, 2008). A second view, posited by homeostatic theories, assumes there is an optimal level of stimulation for an individual and that stereotypical motor movements serve a compensatory function to increase arousal in under-stimulating environments or reduce arousal in over-stimulating environments (Kinsbourne, 1980). A third sensory-equilibration view contends that individuals with ASD engage in stereotypical movements to modulate auditory, visual, vestibular, tactile, and/or proprioceptive stimulation (Gabriels et al., 2008) by dampening sensory stimulation or by inducing heightened sensory experience. Another approach is to view stereotypies as a *motor disorder* that does not depend on functional interpretation, but reflects involuntary output of a dysregulated motor control system, likely including the basal ganglia and dopaminergic pathways (Graybiel, 2008; Langen et al., 2011). To date, the pathophysiology of stereotypies remains undefined (Lewis and Kim, 2009; Langen et al., 2011). The highly diverse phenomenology of this behavior is an additional challenge for developing research programs to address its neurophysiologic basis. Limited effective behavioral management approaches and pharmacological treatment are direct consequences of this lack of understanding.

As a prerequisite to studying their physiology, we felt compelled to develop a classification based on our systematic characterization from video scoring (see Table 1). In a previous study, we reported the prevalence and characteristics of motor stereotypies in developmentally impaired preschool children with and without autism, using the scoring system we have developed (Goldman et al., 2009). Our approach was purely phenomenological and we purposefully kept away from any type of interpretation such as their putative function. We developed a fine-grained video coding to assist identification and provide clinicians and researchers with a systematic descriptive approach for classifying these disparate movements. This video coding has been applied in clinical (LeMonda et al., 2012) and imaging (Goldman et al., 2013) studies. Herein, we present a collection of videos to illustrate both the similarities and the variability of stereotypies observed in children with autism and how some of them evolve over time.

**Table 1:** **Types of stereotypies**.

**Body parts**	**Types of movements**
Face	Grimacing, lips, tongue movements, opening the mouth
Head, trunk, shoulders	Head tilting, shaking, nodding; body rocking, bending, crunching; arching the back; shrugging the shoulders
Arm/leg	Flapping, crossing the arms on the chest, stamping the feet
Hand/finger	Shaking, tapping, waving, clapping, opening-closing, twirling the hand or fingers
Hand/finger with object	Shaking, tapping, twirling an object
Gait	Pacing, jumping, running, skipping, spinning
Self-directed	Covering the ears; mouthing; smelling; rubbing the eyes; tapping the chin; banging the arms against the body; slapping self or an object or surface; touching genitals
Visual	Staring at an object or the fingers “out of the corner of the eyes”

Reproduced with permission from Wiley-Blackwell.

## Methods

To define the spectrum of expression of stereotypies we undertook to study over 500 videos of standardized play sessions recorded between 1985 and 1992 as part of a multi-center, multidisciplinary, longitudinal, nosological study of children with autism, and other developmental disorders (Rapin, 1996). The patients and methods were described previously (Goldman et al., 2009). Briefly, we examined the videotapes of semi-structured play session of preschool children diagnosed with DSM–III-R (American Psychiatric Association, 1987) Autistic Disorder (AD or classical autism) (Rapin, 1996). Children were engaged in play using a uniform set of representational toys (e.g., cars, block, crayons, ball, and puppets). Examiners at four sites: Boston, MA; the Bronx, NY; Cleveland, OH; and Trenton, NJ were trained to interact and play with the children in a similar way. This paper focuses on the 129 children with AD recruited at preschool, of whom 76 were re-examined at age 7 years, and 77 at age 9 years. The mean age at recording was 4.5 years (54 months) (age range 35–97 months). Children with autism were divided in two groups based on nonverbal IQ (NVIQ): high functioning autism (HFA) with NVIQ ≥ 80 and low functioning autism (LFA) with NVIQ < 80. Most of the children shown here were in the LFA group because, overall, this group exhibits higher numbers of stereotypies and thus has most teaching value. All clips were edited from the 30 min-play sessions. We identified as stereotypy any apparently purposeless movement seen at least twice to document its repetitive nature (Goldman et al., 2009). The video clips presented in this paper were selected for their didactic quality in order to best illustrate the *variety* of motor stereotypies in autism.

All the parents of children in the original study gave written informed consent approved by the Institutional Review Board of each institution to videotape their children for research. Parents of Bronx children whose videos are presented in this report signed an additional permission approved by the Institutional Review Board of the Albert Einstein College of Medicine for their children's videos to be shown at scientific meetings and included in scientific publications.

## Illustration and legends to the videos

We selected from our video collection the following 32 clips to demonstrate the variety and co-occurrence of aberrant repetitive motor behaviors observed in our large cohort of children with autism. The segments are organized into eight categories (Movies 1 and 2). For clarity, children are numbered within each category. When available we present video clips of the same child over time to illustrate the evolution and also the constancy of a particular stereotypical movement over time. In order to document the qualitative differences among apparently similar movements usually lumped together under the common “stereotypy” label we present here several clips of the same stereotypy in different children.

### Movie 1

**1: Gait.** These locomotion movements were all potentially normal movements but performed repetitively.

Segment #1: Child #1 circles endlessly around a table and cannot be distracted.

Segments #2, 3: Child #2 is seen at preschool spinning around a spot on the floor and then stamping.

Segment #4: Child #2 circles in place 5 years later. The hint of dystonic arm abduction seen in preschool has become a more obviously dystonic hand posturing with finger twiddling in this later clip.

Segment #5: Child #3 engages in brief complex movements involving jumping with arm movements and vocalizations.

**2: Head trunk and shoulders.** These axial movements were almost always rhythmic. These repetitive behaviors might be considered normal, but were performed beyond what might be considered a typical duration.

Segment #6: Child #1 shows repetitive head nodding.

Segment #7: Child #2 has sustained abnormal head position. Tonic positions, such as the neck extension in Child #2, were less common than repetitive movements in neck, trunk, and limbs.

Segment #8: Child #3 shows more complex axial movements.

Segment #9: Child #4 illustrates truncal rocking, sideways and back and forth.

**3: Arm/hand/finger without objects.** These types of movements, such as flapping, were the most common in the youngest group. The hand movements could be unilateral or bilateral.

Segments #10–12: Children #1, #2, and #3 are examples of clapping movements. Child #2 presented with clapping, leg swinging accompanied by vocalizations.

Segments #13–15: Children #3, #4, and #5 illustrate a common movement involving rapid shaking of the limb around a limp wrist (or elbow), which we have called twiddling. Both Child #1 in segment #10 (clapping and tonic head extension) and Child #3 in this segment (twiddling and tonic finger posturing) combined rhythmic and tonic movements.

Segments #16–17: Children #6 shows a brief rotation movement of the wrist and child #7 a unilateral more continuous shaking.

**4: Hand/finger with objects.** These movements involved repetitive, patterned manipulation of an object or part of an object. Some of these movements were similar to movements without objects described in the previous category, such as clapping objects together instead of clapping hands together. In other movements, the use of the object was an integral part of the movement (especially a peculiar form of playing with objects we call cluttering).

Segments #18, 19, 20: Child #1 illustrates rapid cluttering with objects at preschool and 4 years later. Child #2 clutters in a different more rhythmic way and with dystonic posturing of the thumbs.

Segment #21: Child #3 shakes objects with either hand.

Segment #22: Child #4 claps objects together.

Segment #23: Child #5 rolls objects.

### Movie 2

**5: Self-directed movements.** These self-touching, repetitive movements in which children touched or hit themselves were especially common in LFA children and can be self-injurious. Segments #24, 25: Child #1 brings his face to the object while both Child #1 and Child #2 bring objects to their faces.

Segments #26, 27: Child #3 obsessively rubs his nose at ages 7 and 9, with either hand.

Segment #28: Child #4 claps and picks at his fingers.

**6: Sustained posturing.** Posturing stereotypies are characterized by short episodes of sustained dystonic posturing in the upper or lower limbs.

Segments #29, 30: Child #1 is a low functioning girl with autism seen at preschool and 5 years later. She exhibits a variety of briefly sustained postures, purely repetitive movements, and combinations of posturing with superimposed repetitive movements.

**7: Complex movements.** These more complex stereotypies combined motor and vocal repetitive patterned behaviors.

Segment #31: Child #1 is an example of a complex motor/vocal stereotypy.

**8: Persistence of complex movements over time.** This clip exemplifies the persistence over time of a very particular stereotyped movement which becomes the signature stereotypy for this child.

Segment #32: Child #1 shows a self-hugging stereotypy at ages 4 and 9.

## Conclusion

Only a few studies have characterized stereotypies in detail (Symons et al., 2005). Our collection of 129 preschool children with a DSM–III-R (American Psychiatric Association, 1987) Autism Disorder diagnosis (the more severe classical form of autism) provided the opportunity to describe in depth each stereotypy observed over a fixed time interval (15 min) under standard play conditions (Goldman et al., 2009). The present report and the accompanying video clips illustrate the clinical variability of stereotypies in children with autism. We demonstrate that frequency, rhythmicity, tone, topography, and especially variety of movements can be characterized from video clips and can distinguish subgroups.

So far the main techniques for identifying the presence and the frequency of stereotypies are questionnaires/ interviews of parents/caretakers. These instruments allow for larger data collection and broader contexts, including age and cognitive ability. Direct observation (ideally from videos) and small sample case studies (DiGennaro Reed et al., 2012; Honey et al., 2012) provide detailed analysis and have often been limited to the study of environmental triggers. Despite advances in the validation of these instruments, none of them provides the necessary detailed description required for a differential diagnosis. Indeed, very few studies have developed methods for video coding to examine features of distinct stereotypies and compare them with the abnormal movements of other developmental disorders. Using this video-based clinical approach, Goldman and Temudo (2012) were able to identify striking differences in hand stereotypies of children with ASD and Rett syndrome (RTT) which prove to be important clinical signs for the differential diagnosis of RTT vs. ASD, especially when genetic testing for RTT is not an option.

A small number of longitudinal studies focus on the trajectories of stereotypies (Wetherby et al., 2004; Honey et al., 2006; Esbensen et al., 2008) and present relevant data about diagnostic outcome. For example, longitudinal evaluation of the younger at risk siblings of children with autism suggests that repetitive patterned behaviors may be early diagnostic markers for autism (Gamliel et al., 2007; Rogers, 2009; Zwaigenbaum et al., 2009). So far we are not aware of studies reporting data on trajectories of specific types of stereotypies.

Based on our video-analysis we found that among the 129 preschool children with AD the prevalence of stereotypies in the low functioning autism (LFA) group was 70.6%, marginally statistically higher than the 63.6% found in the high functioning autism group (HFA). In the larger cohort of 277 children that included children with autism and non-autism developmental disorders, statistical analysis comparing the autism to non-autism and cognitively competent to less competent (non-verbal IQ < 80) groups showed that the presence of stereotypies at preschool in the particular setting of our standardized play session was more strongly linked to autism than to cognitive incompetence. Moreover, the number of stereotypies per child and the variety of stereotypies was higher in the LFA group, with head/trunk, hand/finger, and gait (e.g., spinning, pacing) stereotypies being the most frequent in this group (Goldman et al., 2009).

Yet, our longitudinal observation shows that when stereotypies persist, they tend to remain essentially unchanged, at least over a period of several years (in preparation). The fact that the same involuntary movement is produced under a great variety of circumstances and over a long time span lends strength to the hypothesis that specific neuronal circuitry may be responsible for that particular stereotypy (i.e., *the motor disorder* view). Our follow-up clinical observational using objective computerized quantitative measures (i.e., body sensors, accelerometers) of the frequency, topography, complexity, duration, and amplitude of stereotypies in relation to electrophysiological and brain imaging measures are addressing this hypothesis. Another important factor that requires attention pertains to the potential effect of familiarity and context such as home vs. school or the laboratory.

We predict that our video-phenomenology-based approach will prove useful to clinicians and researchers to refine their observation and the analysis of the trajectory of repetitive movements as a function of age, cognitive ability and diagnosis. As such, we also believe that the kind of video-scoring that we have developed and that we discuss here may be instrumental in assessing efficacy of the treatments, which questionnaires or rating forms cannot provide reliably.

### Conflict of interest statement

The authors declare that the research was conducted in the absence of any commercial or financial relationships that could be construed as a potential conflict of interest.
